# Postbiotic Preparation of *Lacticaseibacillus rhamnosus GG* against Diarrhea and Oxidative Stress Induced by Spike Protein of SARS-CoV-2 in Human Enterocytes

**DOI:** 10.3390/antiox12101878

**Published:** 2023-10-19

**Authors:** Marco Poeta, Valentina Cioffi, Antonietta Tarallo, Carla Damiano, Andrea Lo Vecchio, Eugenia Bruzzese, Giancarlo Parenti, Alfredo Guarino

**Affiliations:** 1 Department of Translational Medical Sciences, Section of Pediatrics, University of Naples Federico II, 80138 Naples, Italy; 2 Telethon Institute of Genetics and Medicine, 80078 Pozzuoli, Italy

**Keywords:** COVID, diarrhea, gastroenteritis, *Lacticaseibacillus rhamnosus GG*, oxidative stress, probiotics, postbiotics, SARS-CoV-2

## Abstract

The Spike protein of SARS-CoV-2 acts as an enterotoxin able to induce chloride secretion and production of reactive oxygen species (ROS), involved in diarrhea pathogenesis. *L. rhamnosus GG* (LGG) is recommended in pediatric acute gastroenteritis guidelines as a therapy independent of infectious etiology. We tested a postbiotic preparation of LGG (mLGG) in an in vitro model of COVID-associated diarrhea. Caco-2 cell monolayers mounted in Ussing chambers were exposed to Spike protein, and electrical parameters of secretory effect (*Isc* and TEER) were recorded in the Ussing chambers system. Oxidative stress was analyzed by measuring ROS production (DCFH-DA), GSH levels (DNTB), and lipid peroxidation (TBARS). Experiments were repeated after mLGG pretreatment of cells. The *Isc* increase induced by Spike was consistent with the secretory diarrhea pattern, which was dependent on oxidative stress defined by a 2-fold increase in ROS production and lipid peroxidation and variation in glutathione levels. mLGG pretreatment significantly reduced the secretory effect (*p* = 0.002) and oxidative stress, namely ROS (*p* < 0.001), lipid peroxidation (*p* < 0.001), and glutathione level changes (*p* < 0.001). LGG counteracts Spike-induced diarrhea by inhibiting the enterotoxic effect and oxidative stress. The LGG efficacy in the form of a postbiotic depends on metabolites secreted in the medium with antioxidant properties similar to NAC. Because SARS-CoV-2 is an enteric pathogen, the efficacy of LGG independent of etiology in the treatment of acute gastroenteritis is confirmed by our data.

## 1. Introduction

SARS-CoV-2, similar to other coronaviruses, is a virus with an enteric tropism, capable of infecting enterocytes after binding to ACE-2 receptors located on the intestinal apical surface [1], producing different gastrointestinal symptoms, of which diarrhea is the most frequent, especially in pediatric age [2]. Diarrhea is generally mild and characterized by loose or watery stools [3]. In addition, the gut might play a crucial role in generating systemic inflammation in patients with multisystem inflammatory syndrome in children (MIS-C), an age-specific life-threatening complication of COVID, which, in a number of cases, may present with secretory diarrhea, severe dehydration, and hypovolemic shock [2]. Furthermore, SARS-CoV-2 was isolated in the stools of COVID patients even after nasopharyngeal swabs were negative [4], suggesting a prolonged fecal–oral transmission and a role of intestinal viral persistence in the development of gastrointestinal symptoms of Long COVID syndrome [5].

The pathogenic mechanisms of COVID-associated diarrhea are multiple, and the Spike protein plays a key role in secretory diarrhea. Indeed, in addition to allowing the virus to penetrate the enterocyte causing infection [6], the Spike protein induces chloride secretion [7], activates the local inflammatory response [8], and acts as an oxidant agent in human enterocytes [7].

Interestingly, despite being clinically milder than other viral gastroenteritis [3], SARS-CoV-2-induced diarrhea shares similar mechanisms of action with rotavirus. Rotavirus-associated diarrhea is also caused by a combination of secretory and osmotic mechanisms. The active chloride secretion is triggered by the enterotoxin NSP4, which induces the opening of a calcium-activated chloride channel (CaCC) expressed on the apical surface of enterocytes and anion secretion acting as the Spike protein of SARS-CoV-2 [7]. This enterotoxic effect depends on oxidative stress, which leads to an increase in the intracellular calcium concentration and subsequent activation of CaCC [7]. Therefore, SARS-CoV-2 should be considered an enteric virus responsible for acute gastroenteritis. Whereas the rotavirus replication in intestinal cells induces epithelial damage, reduction in the digestive–absorptive surface, and passive water flux through an osmotic mechanism driven by unabsorbed molecules in the gut lumen, we found no cytopathic effects induced by the Spike protein as judged by TEER experiments; of note, cytopathic damage was induced by infecting Caco-2 cells with the living virus instead of by exposure to pure Spike protein [7].

Several guidelines recommend the use of specific probiotics as adjunctive active treatment of acute gastroenteritis [9]. In vitro and in vivo studies indicate that probiotics produce antidiarrheal effects by inducing changes in microbiota composition and exerting anti-inflammatory effects [10]. However, the antidiarrheal effect observed in children is rapid and already evident in hours and almost always in less than one day, whereas the change in microbiome composition requires prolonged probiotic treatment. Therefore, the change in the microbiome is not the main antidiarrheal mechanism of probiotics, and the rapid effect against diarrhea may be due to the presence of secreted molecules that act directly on intestinal epithelial cells. Similar considerations apply to the changes in immune response induced by selected probiotic strains effective against infectious diarrhea [11].

The hypothesis of a direct effect of probiotics against diarrhea is supported by our recent in vitro study that tested the efficacy of *Lacticaseibacillus rhamnosus GG* (LGG) against rotavirus-induced diarrhea. We demonstrated that a postbiotic preparation of LGG, instead of the living probiotic, is responsible for the rapid antidiarrheal effect due to the presence of moieties with antioxidant properties produced by LGG and secreted in the culture medium, which hampers rotavirus-induced chloride secretion [12].

In the present study, we investigated the effects of the same postbiotic preparation of LGG (mLGG) against secretory diarrhea, evaluated through the Ussing chambers system, and oxidative stress induced by the Spike protein of SARS-CoV-2 in an in vitro experimental model of human-derived enterocytes.

## 2. Materials and Methods

### 2.1. Cell Line

Caco-2 cells (American Type Culture Collection, Middlesex, UK) derived from human colon carcinoma are cells able to differentiate into enterocytes of the upper villus, forming monolayers, and were previously tested in SARS-CoV-2 infection protocols [13]. The cells (passage: 9–12) were grown in high-glucose (4.5 g/L) Dulbecco’s modified Eagle minimum essential medium (DMEM; Gibco, Thermo Fisher Scientific, Oxfordshire, UK) supplemented with fetal calf serum (FBS) (10%; Gibco), nonessential amino acids (1%), and penicillin-streptomycin (100 U/mL; Gibco). The cells were cultured for 15–18 days on polycarbonate Snapwell filters (pore size 0.4 micron) (Costar Italia, Milan, Italy) with medium changes every 48 h. A commercial Spike protein (RBD [V367F, SPD-S52H4]; ACRO Biosystems, Newark, DE, USA) was added to the apical side of the Caco-2 cell monolayers at different doses (1 ng/mL or 1 μg/mL) selected as the same as those used in previous experiments [7,8].

### 2.2. Preparation of Lacticaseibacillus Culture Supernatant

LGG supernatant (mLGG) was prepared as previously described [12]. LGG (6 × 10^9^ u.f.c.) (Dicofarm S.p.A., Rome, Italy) was cultured for 72 h at 42 °C in DMEM (Gibco, Thermo Fisher Scientific, Oxfordshire, UK) with a high glucose concentration (4.5 g/L) supplemented with 10% fetal bovine serum (FBS, Life Technologies Italia, Monza, Italy) and 1% nonessential amino acids. The medium was centrifugated and filtered through a 0.22 mm filter to obtain a bacteria-free culture supernatant (mLGG).

### 2.3. Ion Transport Studies

The Ussing chamber system is used to evaluate ion transport in polarized cell monolayers. Cells were grown on permeable supports with a filter area of 1.18 cm^2^. Each filter was mounted in a Ussing chamber (Physiological Instruments, San Diego, CA, USA) between the mucosal and serosal compartments, containing on each side 5 mL of Ringer’s solution with the following composition (in mmol/L): 114 NaCl; 5 KCl; 1.65 Na_2_HPO_4_; 0.3 NaH_2_PO_4_; 1.25 CaCl_2_; 1.1 MgCl_2_; 25 NaHCO_3_; and 10 glucose. The buffer was gassed with 95% O_2_ and 5% CO_2_ and connected to a thermostat-regulated circulating pump to maintain a temperature of 37 °C. Caco-2 cells were short-circuited by a voltage clamp, and ion transport was studied by measuring modifications in the intensity of short-circuit current (*Isc*), where an increase indicates active luminally directed anion secretion.

The Ussing chambers system is able to define the secretion and absorption of ions through the measurement of electrical parameters and is widely used to study infectious diarrhea induced by viruses, bacteria, and toxins. The maximal variation of *Isc* (Δ*Isc*) indicates mucosal ion secretion (peak effect), while the area under the curve (AUC) is an expression of the potency of the effect. The increase in *Isc* in response to the serosal addition of theophylline (5 mmol/L) was used to test cell viability at the end of each experiment.

The Spike protein (1 ng/mL) was added to the mucosal side of Caco-2 cell monolayers. Untreated cells mounted in the Ussing chamber were used as controls. To test the effects of the postbiotic preparation, cells were treated with mLGG 1 h at 37 °C before the mucosal addition of the Spike protein and during the whole experiment. Cell pretreatment with N-Acetylcysteine (NAC) (20 mM 1 h at 37 °C) was used as a positive control of the antioxidant ability of mLGG.

### 2.4. Transepithelial Electrical Resistance Measurements

Transepithelial electrical resistance (TEER) of cell monolayers grown on filters (4.9 cm^2^) was measured using a Millicel-ERS resistance monitoring apparatus (Millipore). The value of TEER (in Ohms/cm^2^) was calculated by subtracting the background value. Cell monolayers were treated with the Spike protein (1 μg/mL) in the presence or not of mLGG. TEER was recorded every 24 h up to 72 h. As positive controls, cells were incubated with the toxic agent, Sodium Arsenite (ARS) (300 μM), instead of the Spike protein.

### 2.5. Reactive Oxygen Species (ROS)

ROS production was measured using 79-dichlorofluorescein diacetate (DCFH-DA, D6665; Sigma-Aldrich, St. Louis, MO, USA) spectrofluorometry (SFM 25; Kontron Instruments, Tokyo, Japan) with emission spectra acquired at 488 nm (excitation) and 525 nm (emission). Caco-2 cells were grown in DMEM in 24-well plates for 18 days post-confluence. Cell monolayers were treated with the Spike protein (1 μg/mL) for 15 min at 37 °C as previously described [7] and then with DCFH-DA (20 μM) for 30 min in the dark at 37° C. As positive controls, cells were incubated with the toxic agent, Sodium Arsenite (ARS) (300 μM), instead of the Spike protein. As negative controls, cells were incubated with DCFH-DA in the absence of stimuli. To assess the neutralization potential of mLGG on ROS production, cells were preincubated with mLGG for 1 h at 37 °C before the addition of the Spike protein. As a control of the antioxidant ability of the postbiotic preparation, cells were pretreated for 1 h at 37 °C with NAC (20 mM) instead of mLGG.

### 2.6. Intracellular Glutathione (GSH) Assay

GSH is a key component of antioxidant cell defenses. Intracellular GSH levels were analyzed by using a 5,50—dithiobis-2-nitrobenzoic acid (DTNB) assay. After treatment with the Spike protein (1 μg/mL) for 1 h at 37 °C, with or without 1 h mLGG pretreatment, cells were collected by trypsinization, centrifuged (1000× *g* for 10 min), and resuspended in RIPA buffer with protease inhibitors. After a 30 min incubation in ice, lysates were centrifuged at 4 °C (14,000× *g* for 30 min). The Lowry assay was used to determine supernatant protein concentration. Both 3 mM ethylenediaminetetraacetic acid (EDTA) and 144 µM DTNB (Sigma-Aldrich, St. Louis, MO, USA) were incubated with 50 µg of proteins in a 30 mM Tris–HCl buffer, pH 8.2, and centrifuged at room temperature (14,000× *g* for 5 min). A multiplate reader (Epoch BioTek, Winooski, VT, USA) was used to read absorbance at 412 nm. As a positive control, cells were treated with ARS (300 μM) instead of the Spike protein. As a control of the antioxidant ability of the postbiotic preparation, cells were pretreated with NAC (20 mM) instead of mLGG.

### 2.7. Lipid Peroxidation Assay

Since lipids are susceptible to oxidation and lipid peroxidation products are potential markers for oxidative stress, lipid peroxidation was analyzed using the thiobarbituric acid reactive substances (TBARS) assay. After treatment with the Spike protein (1 mcg/mL) for 1 h at 37 °C, with or without 1 h mLGG pretreatment, cells were collected after trypsinization and centrifuged (1000× *g* for 10 min), and 5 × 10^5^ cells were resuspended in 1 mL of 0.67% thiobarbituric acid (TBA) (SigmaAldrich, St. Louis, MO, USA). After the addition of 300 μL, 20% trichloroacetic acid (TCA) (Sigma-Aldrich, St. Louis, MO, USA) samples were heated at 95 °C for 30 min, incubated in ice for 10 min, and centrifuged at 4 °C (3000× *g* for 5 min). A multiplate reader (Epoch Biotek Winooski, USA) was used to read absorbance at 532 nm. As a positive control, cells were treated with ARS (300 μM) instead of the Spike protein. As a control of the antioxidant ability of the postbiotic preparation, cells were pretreated with NAC (20 mM) instead of mLGG.

### 2.8. Statistical Analysis

GraphPad Prism Software (Version 6.01, San Diego, CA, USA) was used to evaluate the two-tailed unpaired Student t-test. An alpha value of 0.05 was set for statistical significance. *p*-values for each analysis are indicated in legends of figures.

## 3. Results

### 3.1. Effects on Chloride Secretion

**Figure 1:**
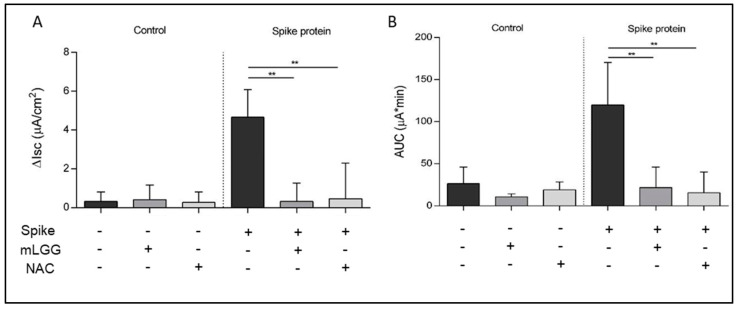
Effects of mLGG on enterotoxic effect induced by Spike protein of SARS-CoV-2 in Caco-2 cell monolayers. (**A**) Spike protein induced an increase in the intensity of short-circuit current (*Isc*), which was prevented by preincubation of Caco-2 cells with mLGG or NAC as described in Methods. (**B**) The potency of the effect (assessed as the AUC) shows similarity between mLGG and NAC. **Abbreviations:** AUC, area under the curve; CTRL, control cells; *Isc*, intensity of short-circuit current; NAC, N-acetylcysteine; ** *p* < 0.0005.

As previously reported in the basic model of Caco-2 cells [7], the mucosal addition of the Spike protein induced a statistically significant increase in *Isc* in the Ussing chambers experiments, indicating electrogenic fluid secretion (Figure 1). Effects on *Isc* were observed only when Spike was added to the mucosal but not the serosal side of the Caco-2 cell monolayers [7].

To investigate the effects of mLGG on Spike-induced chloride secretion, Caco-2 cell monolayers were preincubated with mLGG, and subsequently, Spike was added to the mucosal side of intestinal cells. The magnitude of the short-circuit current increase (Δ*Isc*), which reflects the intensity of Spike-induced chloride secretion, was significantly reduced by mLGG (Spike 2.9 ± 0.3 µA/cm^2^ vs. mLGG + Spike 0.9 ± 0.32 µA/cm^2^; *p* = 0.002) (Figure 1A). mLGG alone did not induce Isc changes.

Since the Spike-induced enterotoxic effect is oxidative-stress-dependent [7], we preincubated Caco-2 cells with NAC instead of mLGG, resulting in the prevention of chloride secretion in terms of *Isc* variations with a similar potency of mLGG as indicated by the AUC data (Figure 1B).

**Figure 2:**
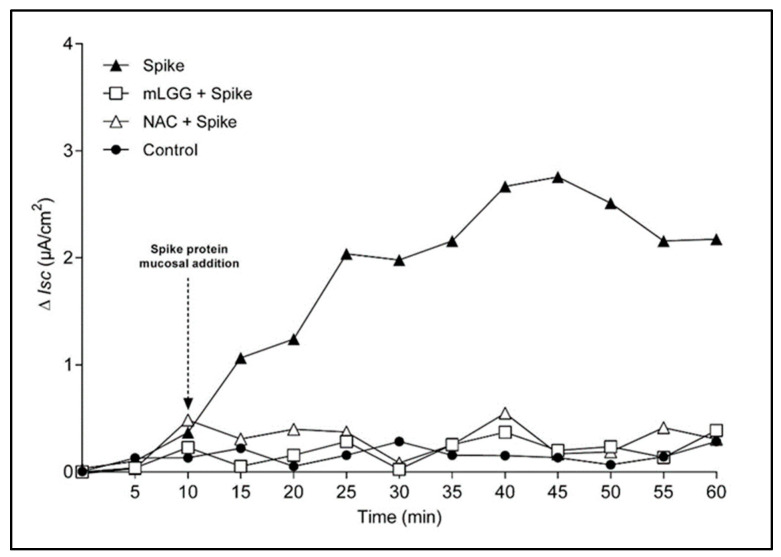
Time course analysis of Spike-induced electrogenic effect in Caco-2 cells. *Isc* was measured every 5 min for 60 min. Cells were stimulated at 10 min with Spike protein (arrow), with or without 1 h preincubation with mLGG or NAC. Untreated cells were used as controls. mLGG and NAC pretreatment of cells prevented Spike-induced electrogenic changes from the start of the experiment. **Abbreviations:** *Isc*, intensity of short-circuit current; NAC, N-acetylcysteine.

The effect was evident 15–20 min after the addition of the Spike protein, reaching a peak at 40–45 min, after which the *Isc* value remained stable (Figure 2). Preincubation with mLGG or NAC completely prevented the enterotoxic effect from the start of the experiment.

### 3.2. Effects on Epithelial Integrity

**Figure 3:**
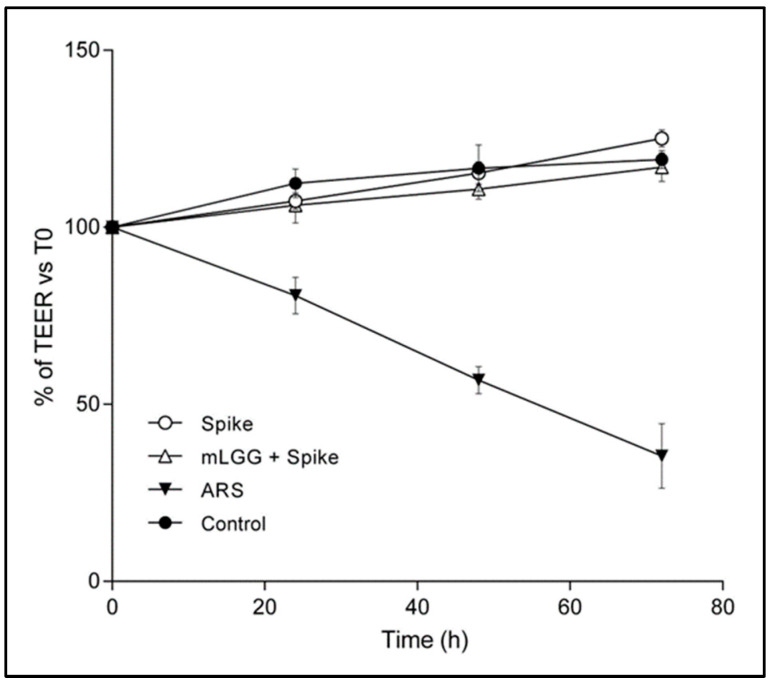
Transepithelial electrical resistance in Caco-2 cells following addition of Spike protein. Cell monolayers were exposed to Spike protein at the mucosal side, with or without mLGG. Untreated and ARS-treated cells were used as negative and positive controls, respectively. TEER was recorded every 24 h up to 72 h. No changes were recorded after Spike protein addition. mLGG did not induce TEER decrease, reflecting the absence of cytotoxic effect of the postbiotic preparation. **Abbreviations:** ARS, sodium arsenite; T0, TEER prior to exposure; TEER, transepithelial electrical resistance.

A reduction in transepithelial electrical resistance (TEER) indicates a disruption of epithelial integrity [12]. As in previous experiments [7], pure Spike protein did not induce a decrease in TEER over 72 h following exposure. The pretreatment of cells with mLGG did not induce TEER changes, supporting the safety of the postbiotic preparation, which had no cytotoxic effects on enterocytes (Figure 3).

### 3.3. Effects on ROS Production

ROS production was evaluated in Caco-2 cells exposed to the Spike protein with or without mLGG preincubation. The Spike protein induced a two-fold increase in ROS production, and the effect was observed 15 min after exposure to the protein (*p* < 0.0005), similar to what happened with the oxidant agent ARS, which induced a four-fold increase (*p* < 0.0005).

**Figure 4:**
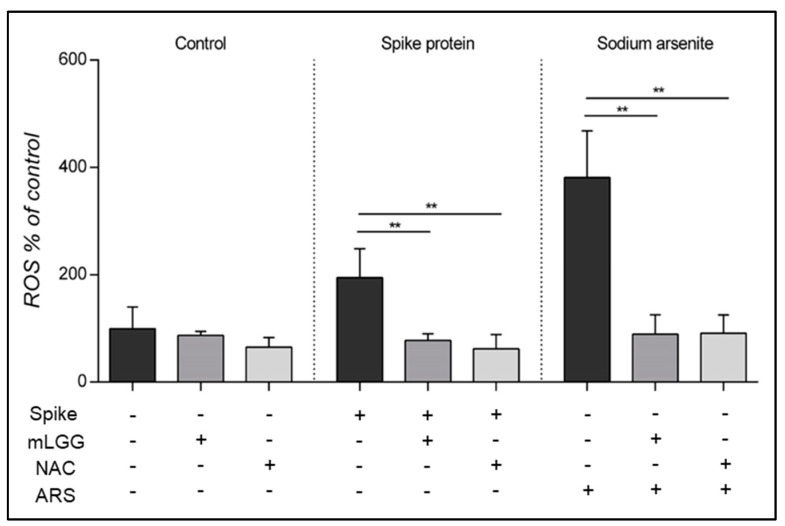
Effects of mLGG on Spike-induced ROS production in Caco-2 cells. Intracellular ROS levels were evaluated using DCFH-DA fluorometry 15 min after exposure to Spike protein (1 μg/mL) with or without 1 h preincubation with mLGG or NAC. Untreated cells and ARS-treated cells were used as negative and positive controls, respectively. mLGG prevented ROS production induced by both Spike protein and ARS, with effects similar to NAC. **Abbreviations:** ARS, sodium arsenite; CTRL, control cells; NAC, N-acetylcysteine; ROS, reactive oxygen species; ** *p* < 0.0005.

ROS production was completely prevented by mLGG similar to NAC. mLGG was also able to prevent ROS production induced by ARS (Figure 4).

### 3.4. Effects on GSH Levels

To evaluate the role of antioxidant defenses, GSH was measured after Spike exposure with or without mLGG preincubation of cells. A reduction in GSH levels was observed after Spike protein (*p* = 0.0002) or ARS exposure (*p* < 0.0001).

**Figure 5:**
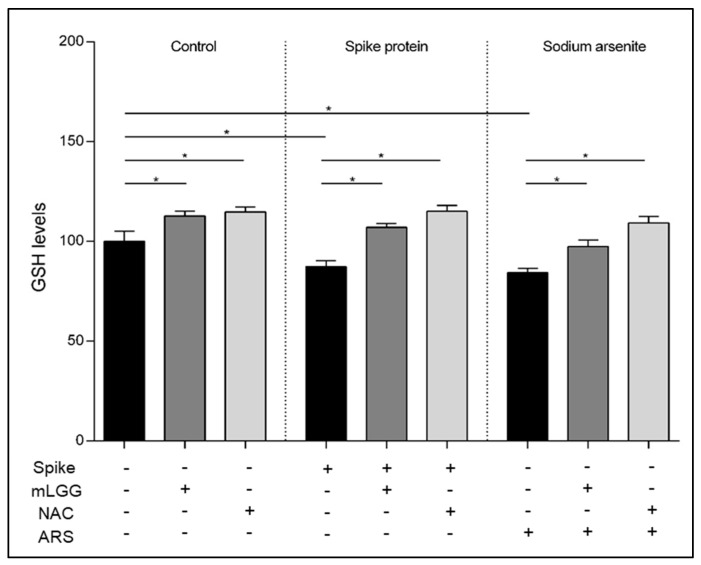
Effects of mLGG on GSH levels. GSH was evaluated 1 h following Spike protein exposure of Caco-2 cell monolayers with or without mLGG preincubation. NAC was used as control of antioxidant ability of mLGG. Untreated and ARS-treated cells were used as negative and positive controls, respectively. mLGG prevented the reduction in GSH levels induced by both Spike protein and ARS, with effects similar to NAC. **Abbreviations:** ARS, sodium arsenite; CTRL, control cells; GSH, reduced glutathione; NAC, N-acetylcysteine. * *p* < 0.0005.

mLGG preincubation resulted in an antioxidant preventive effect, similar to NAC, reverting GSH levels to control cell values (Figure 5).

### 3.5. Effects on Lipid Peroxidation

To assess oxidative status, in addition to ROS and GSH levels, lipid peroxidation was evaluated in Caco-2 cells after exposure to Spike protein. A 3.5-fold increase in lipid peroxidation was found after Spike protein treatment compared to control cells (*p* < 0.001), with an effect higher than that induced by the oxidant agent ARS (2-fold increase).

**Figure 6:**
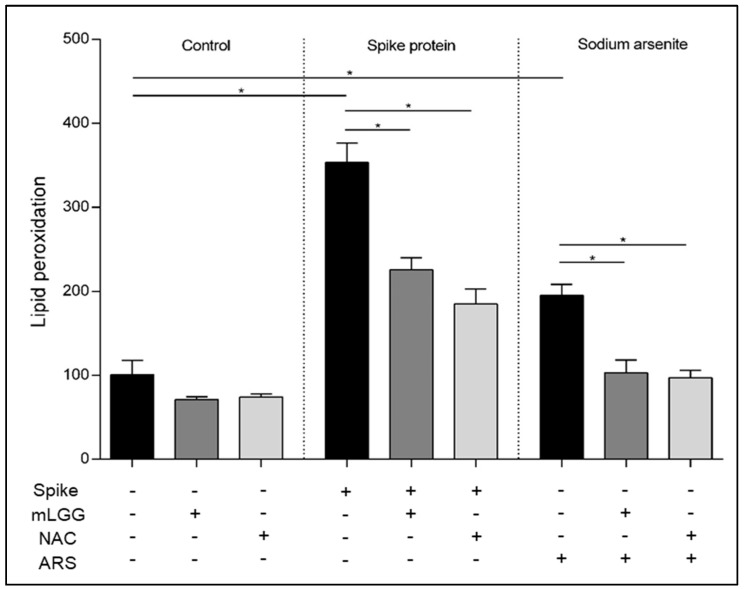
Effects of mLGG on lipid peroxidation. Lipid peroxidation was evaluated 1 h following Spike protein exposure of Caco-2 cell monolayers with or without mLGG preincubation. NAC was used as control of antioxidant ability of mLGG. Untreated and ARS-treated cells were used as negative and positive controls, respectively. mLGG prevented lipid peroxidation induced by both Spike protein and ARS, with effects similar to NAC. **Abbreviations:** ARS, sodium arsenite; CTRL, control cells; NAC, N-acetylcysteine. * *p* < 0.0005.

mLGG preincubation prevented lipid peroxidation induced by both Spike and ARS, similar to what happened with NAC pretreatment (Figure 6).

## 4. Discussion

SARS-CoV-2 has been recognized as a novel agent of acute gastroenteritis. After infection of intestinal cells, patients with COVID may present a number of gastrointestinal symptoms, of which diarrhea is the most common. Although the clinical course of COVID-associated diarrhea is mild [3], the presence of this symptom is often associated with a more severe COVID course in terms of local and systemic inflammatory response, making diarrhea a hallmark of systemic inflammation and the gut a key organ involved in the development of the so-called “cytokine storm” [3,14].

The present work confirms that the spike protein of SARS-CoV-2 is an enterotoxin with oxidant capabilities as demonstrated by the increase in ROS [7], adding experimental evidence regarding lipid peroxidation and intracellular GSH levels in human intestinal cells. The ROS increase was previously reported in endothelial and respiratory cells after SARS-CoV-2 infection, suggesting a role of oxidative stress in the development of acute respiratory distress syndrome and multiple organ failure in acute COVID [15]. The imbalance between the production of free radicals and antioxidant systems also plays a crucial role in determining COVID severity, and, since the beginning of the pandemic, the administration of NAC (oral, nebulized, or intravenous) has been proposed as a preventive therapy of severe forms [16].

Oxidative stress has direct implications in both osmotic and secretory diarrhea mechanisms induced by SARS-CoV-2. In particular, it is known that ROS can act either as mediators of inflammation or as signaling molecules [17]. Hence, ROS are implicated both in the damage of the intestinal epithelium, with the consequent development of osmotic diarrhea, and as a trigger of secretory diarrhea, inducing the increase in intracellular calcium concentration and the consequent CaCC opening and chloride secretion [18]. Furthermore, considering that the increase in intestinal ROS is rapid, reaching a peak within 15 min after exposure to the Spike protein, oxidative stress appears to be one of the primary mechanisms involved in SARS-CoV-2-induced diarrhea [7]. Moreover, the involvement of oxidative stress in anion secretion is confirmed by the suppression of *Isc* changes after the treatment of cells with NAC, an effective natural antioxidant.

The main therapy of pediatric acute gastroenteritis is the administration of an oral rehydration solution [19], but this does not reduce the duration of diarrhea or the frequency of stools. The administration of selected probiotic strains, including LGG and Saccharomyces boulardii, is proposed as an active treatment as an adjunct to rehydration to reduce the intensity and duration of diarrhea [19]. The proposed mechanisms of action for the antidiarrheal effects of probiotics include the restoration of microbiota, the secretion of antimicrobial substances, the competitive exclusion of pathogens, and the modulation of local and systemic immune response [20,21]. All of these mechanisms require hours or even days to be active and affect the clinical course of acute gastroenteritis [22]. The administration of effective probiotic preparations produces significant results within a few hours from its start [23].

In a recent transcriptomic study on the effect of LGG and L. plantarum on oxidative stress, both strains were able to prevent oxidative stress by slowing down the decrease in superoxide dismutase (SOD) and the increase in glutathione peroxidase (GPX) activity, alleviating oxidative damage in Caco-2 cells [24]. The authors of this study suggested two possible mechanisms: 1. the presence of antioxidant molecules in the bacteria, which scavenge ROS around the cells, and 2. the regulation of pathways associated with antioxidant enzymes in host cells. Furthermore, the antioxidant effect was not found using inactivated bacteria, probably because the antioxidant components lose their activity as part of the inactivation process. In our model, we did not use inactivated bacteria but a bacteria-free culture supernatant (mLGG) obtained through centrifugation and filtration, which did not contain inactivated bacteria or bacterial components but only metabolites secreted in the medium.

We previously demonstrated that the effect of LGG against rotavirus-induced diarrhea depends on the “postbiotic effect” of substances secreted by the bacteria in the culture medium, which appears to have a rapid mechanism of action, more likely consistent with a “pharmacological” action rather than a microbiological effect, eventually resulting in immune stimulation [12]. These substances contained in the postbiotic preparation have antioxidant properties similar to NAC, which are implicated in the rapidity of the effect acting on the first step of diarrhea pathogenesis (i.e., prevention of ROS production). Furthermore, animal studies conducted on mice and piglets demonstrated the antioxidant capabilities of LGG against intestinal and kidney injuries induced by Giardia [25] and mycotoxins [26], respectively. Furthermore, animal studies investigating liver toxicity induced by alcohol [27] or titanium dioxide nanoparticles [28] showed the efficacy of LGG administration in restoring the expression of antioxidative stress-related genes (i.e., SOD1, SOD2, and GSH).

Considering the presence of similarities between rotavirus and SARS-CoV-2-associated diarrhea pathogenesis, in the present work, we demonstrated that LGG in the form of a “postbiotic” is also effective against COVID-associated diarrhea by reducing Spike-induced ion secretion, with an action dependent on the prevention of oxidative stress. Specifically, mLGG was able to prevent electrogenic secretion, as judged by Ussing chamber experiments, with a complete inhibition of *Isc* variation as indicated by the time course experiment. The rapidity of action appeared to be related to the prevention of oxidative stress, considering that similar results were obtained with NAC, a potent antioxidant. Furthermore, the pretreatment of cells with the postbiotic preparation prevented ROS production and lipid peroxidation, restoring normal GSH levels. Considering that oxidative stress plays a crucial role in innate immunity involved in COVID pathogenesis [29], the antioxidant properties of mLGG could reduce the local inflammatory response in the gut, which characterizes acute COVID and is involved in the systemic cytokine storm and progression to severity [3].

In addition, we did not observe modifications in TEER values of Caco-2 cell monolayers after exposure to the Spike protein, confirming the absence of measurable Spike-induced damage to the epithelium, although a mucosal injury induced by the living virus cannot be excluded. However, mLGG did not decrease epithelial electrical resistance up to 72 h of treatment, suggesting the safety of this product in terms of cytotoxicity.

Finally, we tested in all experiments high concentrations (300 μM) of the toxic agent ARS, as a positive control, which was able to induce a significant increase in intracellular ROS production and lipid peroxidation, resulting in reduced GSH. Interestingly, our results confirm the high antioxidant properties of mLGG, which was able to prevent all of the oxidative effects induced also by ARS. The antioxidant properties of the postbiotic preparation appear independent of the oxidant stimuli, in agreement with the results of a transcriptomic in vitro study showing the downregulation of oxidative stress genes in Caco-2 cells treated with LGG [24].

Our study adds information on a potential neutralization effect derived from metabolites secreted by LGG in the culture medium, which could exert antisecretory and antioxidant activities. The main limitation of our study is the absence of other Lactobacillus strains used as controls; in fact, we cannot exclude that these effects are unspecified and shared with other Lactobacillus. Further studies are needed to characterize the composition of mLGG and identify metabolites secreted by bacteria in order to define the potential development of derived antidiarrheal drugs.

## 5. Conclusions

LGG counteracts Spike-induced diarrhea by inhibiting enterotoxic effects and oxidative stress in human enterocytes. The efficacy of LGG in a postbiotic form depends on molecules secreted into the bacteria-free culture medium with antioxidant abilities. Since SARS-CoV-2 is a novel agent of pediatric gastroenteritis, our data confirm the efficacy of LGG regardless of etiology as an active therapy to reduce the intensity and duration of diarrhea. Further clinical studies are needed to define clinical efficacy. Finally, our study contributes to a better understanding of the mechanism of viral diarrhea by highlighting the role of oxidative stress as a major actor in the pathogenesis of secretory mechanisms and as a target of new antidiarrheal drugs.

## Data Availability

The datasets generated and/or analyzed during the current study are available from the corresponding author upon reasonable request.
